# Role Played by Edge-Defects in the Optical Properties of Armchair Graphene Nanoribbons

**DOI:** 10.3390/nano11123229

**Published:** 2021-11-28

**Authors:** Thi-Nga Do, Godfrey Gumbs, Danhong Huang, Bui D. Hoi, Po-Hsin Shih

**Affiliations:** 1 Department of Physics, National Cheng Kung University, Tainan 701, Taiwan; sofia90vn@gmail.com; 2 Department of Physics and Astronomy, Hunter College of the City University of New York, 695 Park Avenue, New York, NY 10065, USA; ggumbs@hunter.cuny.edu; 3 US Air Force Research Laboratory, Space Vehicles Directorate (AFRL/RVSU), Kirtland Air Force Base, Albuquerque, NM 87117, USA; danhong.huang@us.af.mil; 4 Department of Physics, University of Education, Hue University, Hue 530000, Vietnam; buidinhhoi@gmail.com

**Keywords:** defect graphene nanoribbon, absorption function, tight-binding model

## Abstract

We explore the implementation of specific optical properties of armchair graphene nanoribbons (AGNRs) through edge-defect manipulation. This technique employs the tight-binding model in conjunction with the calculated absorption spectral function. Modification of the edge states gives rise to the diverse electronic structures with striking changes in the band gap and special flat bands at low energy. The optical-absorption spectra exhibit unique excitation peaks, and they strongly depend on the type and period of the edge extension. Remarkably, there exist the unusual transition channels associated with the flat bands for selected edge-modified systems. We discovered the special rule governing how the edge-defect influences the electronic and optical properties in AGNRs. Our theoretical prediction demonstrates an efficient way to manipulate the optical properties of AGNRs. This might be of importance in the search for suitable materials designed to have possible technology applications in nano-optical, plasmonic and optoelectronic devices.

## 1. Introduction

Seventeen years have now passed since the discovery of graphene in 2004 [1], and this has unmistakably inspired a huge amount of research on its fundamental properties as well as those of graphene-related materials. Graphene nanoribbons (GNRs), which are narrow strips of graphene, possess quasi-one-dimensional properties. This material presents rich essential physical properties, including electronic, optical, magnetic, and transport properties [2,3,4,5,6,7]. Consequently, they acquire additional advantages over graphene sheets in the point of view of certain technological applications such as nanoelectronics, spintronics, and photodetectors [8,9,10]. Up to now, the bottom-up [11,12,13,14,15] and top-down [16,17,18,19,20] methods have been demonstrated to be efficient for the synthesis of GNRs. It is well known that GNRs can be classified into two categories based on their edge-state arrangement, namely, armchair GNR (AGNR) and zigzag GNR (ZGNR) [21,22]. Both the AGNR and ZGNR can be subjected to edge defects during the fabrication processes, which has been examined to modify remarkably the essential physical properties of the materials. This work will mainly focus on the role played by the edge-extension on the electronic and optical characteristics of AGNRs.

In previous studies, it has been demonstrated that fundamental physical properties of GNRs can be tailored by the presence of edge defects [13,23,24,25], besides edge terminations, ribbon width, vacancy, and applied electrostatic fields [2,3,4,5,26]. The edge-defect GNRs with proper configuration have been demonstrated as being suitable for certain device applications, such as bandgap engineering for generating semiconductor heterostructure devices [12,16], improved electrical conductivity for efficient gas sensing [13], and imbalanced sublattice for creating graphene nanomaterials with magnetically nontrivial ground states [15]. In fact, uncontrolled edge modifications also cause detrimental effects such as transport deterioration and variability, which negatively impact the possibility of using GNRs in nanodevices [27], but it is different from controlled edge defects discussed in this paper. Recently, the band structures and charge-density distribution of AGNRs have been predicted theoretically as well as examined experimentally to be significantly responsive to the kinds of edge modification [23]. Various types of electronic topological phases and the influence of electronic correlations on these topological states have been reported [24,25]. Despite recent progress in this field, the investigation of the optical properties of edge-extended GNRs is still in its infancy. Consequently, engineering the optical spectra of GNRs by introducing edge defect is worthy of a careful investigation.

In this paper, we carefully analyze the electronic and optical properties of AGNRs with various types of edge extensions. The unique absorption spectra and their relations to the special electronic structure, including the tunable band gap and flat bands, will be discussed extensively. The density of states (DOS), which is crucial for an in depth understanding of both the electron state distribution and optical excitation channels, are presented. Our numerical calculations reveal unusual optical selection rules related to the transition of the nearly-flat edge bands. We demonstrate that the frequency and amplitude of the optical-absorption peaks can be efficiently manipulated by both the type and period of the edge extension.

The rest of this paper is organized as follows. In Section 2, we present our theoretical method, which we use for executing our numerical computations of the properties of edge-extended AGNRs. Section 3 contains a detailed discussion of the band structure and absorption spectra for the systems with three different kinds of edge-extension. The principal results of this work are summarized in Section 4.

## 2. Theoretical Method

**Figure 1:**
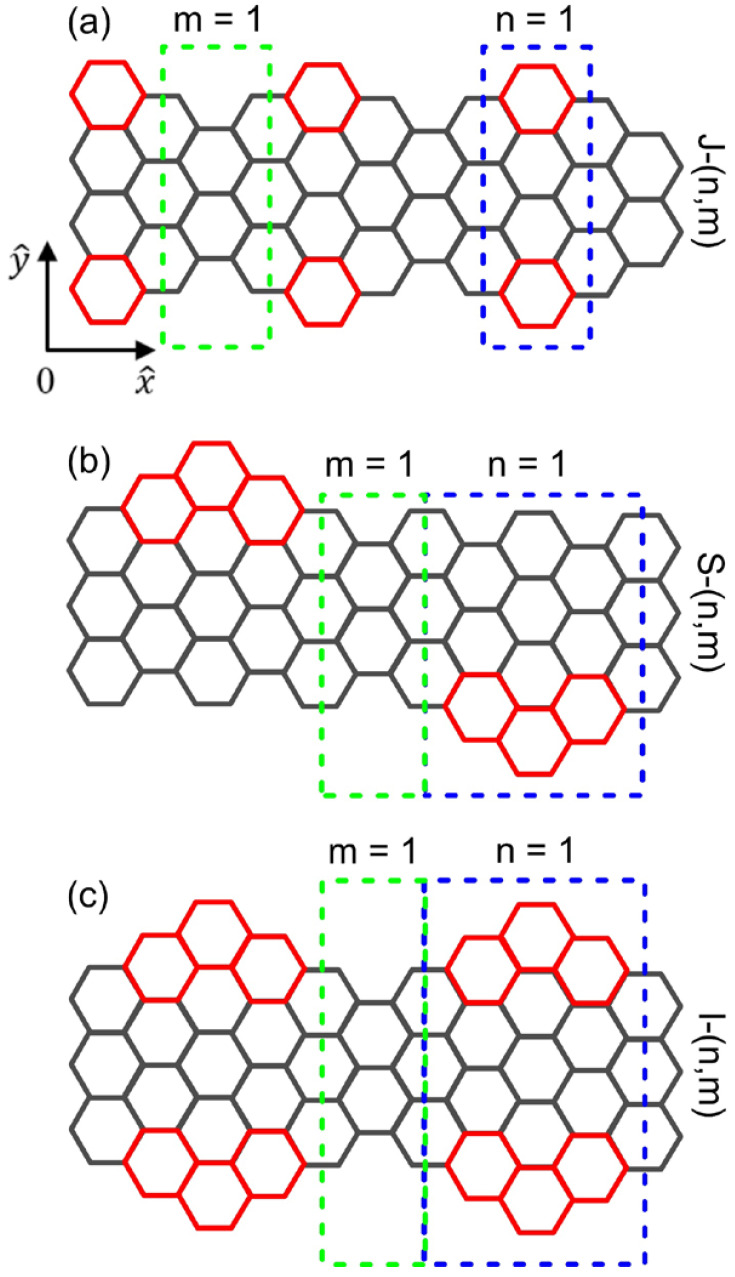
(color online) Lattice structures of edge-extended AGNRs for (**a**) 7/9-AGNR nanoribbon, (**b**) staggered edge-extended AGNR, and (**c**) inline edge-extended AGNR. The grey hexagons denote pristine AGNRs while the red hexagons represent the edge-extended areas. The green and blue dashed rectangles indicate the units of n and m segments, respectively.

We begin by noting that a GNR consists of carbon atoms stacked together to form a honeycomb lattice with a bond length of b=1.42 Å. The tight-binding model (TBM) is employed to investigate the electronic and optical properties of AGNR. The couplings between nearest-neighbor atoms are included in our calculations. For pristine AGNR along the x direction, the width (N) is estimated by the number of carbon atoms across the ribbon along the y axis. Here, we consider the AGNR with extended segments being added periodically on both sides along the x direction. Figure 1a through Figure 1c illustrate, respectively, three different configurations of edge-extended AGNRs; they are 7/9−AGNR heterojunction (J-(n,m)), staggered edge-extended AGNR (S-(n,m)), and inline edge-extended AGNR (I-(n,m)). In this notation, *n* and *m* stand for the length of the edge-extended and original segments, respectively. They are integers in the unit of 3b. A primitive unit cell comprises two continuous *n* and *m* segments. For J-(n,m) and S-(n,m), the two component segments possess the widths of *N* and (N+2), while they are *N* and (N+4) for I-(n,m). Furthermore, the two edges of J-(n,m) and I-(n,m) are equivalent along the backbone of the ribbon. On the other hand, the edge-extended segments are located alternately on each side of the S-(n,m) AGNR. The difference in configuration among these AGNRs gives rise to their distinctive electronic and optical characteristics.

The low-energy electronic properties of an edge-extended AGNR can be described in terms of the $p_{z}$-orbital tight-binding Hamiltonian, which we write as

(1) $$H=\sum_{\langle i,j\rangle }\;\gamma^{R_{ij}}C_{i}^{†}C_{j}+h.c..$$

In this expression, *i* and *j* denote lattice sites, $C_{i}^{†}$ ($C_{j}$) is a creation (annihilation) operator, $R_{ij}$ is the translation vector between two atoms, $\gamma^{R_{ij}}=-2.6\;$eV is the nearest-neighbor atomic interaction [28], and h.c. stands for the Hermitian conjugate of the first term. In general, the hopping term of graphene is defined as the π bond between carbon atoms, which takes the value −3.033 eV [29]. When ones only consider the monorail domain model, this number corresponds directly to the energy at the M point of the first Brillouin zone. However, the energy at the M point of the actual band is usually lower than the expected value due to the influence of other orbital domains. As a matter of fact, the commonly used hopping range of the tight-binding model is [−3.033,−2.4] eV [29,30,31]. This range has been widely adopted to study the electronic structure of GNRs and get a good agreement with experimental observations. We know that the change of hopping interaction can lead to modification of the energy scale of the band structure, but its main features remain unchanged.

This Hamiltonian is properly constructed based on selected lattice sites *i* and *j* of individual AGNR for each edge-extended type to ensure that the boundary conditions are satisfied on a lattice-atomic level. Consequently, the corresponding band structure and wave functions automatically satisfy the boundary condition of the system. Physically, the dominant effect from the edge atomic environment has already been included by employing atomically-satisfied boundary conditions for edge atoms on the lattice. Therefore, the change of bonding strength of edge atoms with respect to interior atoms is considered to be a higher-order correction. In this study, we consider the same hopping interaction for the interior and at the edges of the GNR, but ignore the higher-order effect due to the difference in atomic environment. In this way, we are able to highlight the major effects of edge-extension on the band structure and absorption spectra of armchair GNRs with a narrow width. Moreover, the effect of edge states on the electronic properties of GNRs has been addressed in ref. [32] based on a first-principles (FP) approach. Explicitly, the authors tested the consistency between the FP and (identical hopping integral) TBM results associated with N-AGNR for three different cases: (1) N = 3p (p is a positive integer), (2) N = 3p + 1, and (3) N = 3p + 2. It turned out that the remarkable inconsistency between the two methods only occurs for the case of (3) N = 3p + 2. In other words, the effect of the modified edge-atom integral on the electronic properties becomes significant only for the case with N = 3p + 2. In our present study, we consider the AGNR with the backbone width of N = 7 (corresponding to 3p + 1). Thus, our current TBM with identical hopping integral for edge and interior atoms in the lattice structure is acceptable for the study of electronic properties of the materials.

An electromagnetic field carrying the electric polarization $\hat{E}$ with frequency ω can lead to vertical optical transitions from occupied to unoccupied states in an AGNR. The resulting absorption function for vertical transition and $E_{F}=0$ can be shown to be given by [33]

(2) $$\begin{array}{cc} A(\omega )\propto \; & \sum_{c,v,\alpha ,\beta }\int_{1stBZ}\frac{dk}{{\left(2\pi \right)}^{2}}|\langle \Psi^{c}\left(k,\beta \right)|\frac{\hat{E}\cdot P}{m_{e}}|\Psi^{v}\left(k,\alpha \right)\rangle {|}^{2}\;\\ \; & \times Im\left[\frac{f\left(E^{c}\left(k,\beta \right)\right)-f\left(E^{v}\left(k,\alpha \right)\right)}{E^{c}\left(k,\beta \right)-E^{v}\left(k,\alpha \right)-\omega -i\Gamma }\right]. \end{array}$$

Here, P is the momentum operator, $m_{e}$ is the free-electron mass, $f(E^{c,v}\left(k,\alpha \right))$ is the Fermi–Dirac distribution function, and Γ (= 10 meV) is a broadening parameter. Note that, the broadening factor could be sample dependent. In fact, the broadening factor associated with electron optical transitions results from scattering, including both intrinsic coulomb scattering (important for high density and low temperature) and phonon scattering (important for low density and high temperature), as well as extrinsic impurity and defect scatterings (significant in low-quality samples). In general, Γ = 1 meV, 5–10 meV and much greater than 10 meV correspond to high-, intermedium- and low-quality samples, respectively. Therefore, the selection of Γ = 10 meV in this paper corresponds to an average-quality sample, which is expected to display unique optical features, but not to present in a very-sharp form. It guarantees no overlapping of the peaks so that the main features of the spectra are clearly revealed. Furthermore, this choice of Γ is to be consistent with the reported theoretical calculations and experimental measurements [34,35,36]. Remarkably, the discussed physics mechanism for our current system remains unchanged.

For the absorption function, the available excitation channels are determined by the non-vanishing of the velocity matrix element $\langle \Psi^{c}\left(k,\beta \right)|\frac{\hat{E}\cdot P}{m_{e}}|\Psi^{v}\left(k,\alpha \right)\rangle$. Explicitly, it can be seen from Equation (2) that the absorption coefficient becomes finite only if the velocity matrix element term ($\langle \Psi^{c}\left(k,\beta \right)|\frac{\hat{E}\cdot P}{m_{e}}|\Psi^{v}\left(k,\alpha \right)\rangle$) is nonzero. This indicates that there exist finite interband transitions between initial and final electronic states in different valence and conduction subbands under irradiation of light. Moreover, the velocity matrix element can be estimated based on the gradient approximation of the form [37]

(3) $$\langle \Psi^{c}\left(k,\beta \right|\frac{\hat{E}\cdot P}{m_{e}}|\Psi^{v}\left(k,\alpha \right)\rangle \approx \frac{1}{\hbar }\langle \Psi^{c}\left(k,\beta \right|\frac{\partial H}{\partial k_{x}}|\Psi^{v}\left(k,\alpha \right)\rangle \;.$$

Therefore, the finite transition occurs only if the wave function of an initial valence subband (α) and that of a final conduction subband (β) have the same number of zero modes. This directly leads to the optical selection rule of the system. If the wave functions of initial and final states do not satisfy this selection rule, optical transition between them is forbidden. On the other hand, the absorption strength is proportional to the joint DOS $Im\left[\frac{f\left(E^{c}\left(k,\beta \right)\right)-f\left(E^{v}\left(k,\alpha \right)\right)}{E^{c}\left(k,\beta \right)-E^{v}\left(k,\alpha \right)-\omega -i\Gamma }\right]$.

From a numerical computation aspect, the integration over the *k*-space in Equation (2) can be calculated as a summation over many integrand values in very small subdivided regions of the first Brillouin zone corresponding to different *k* values. Particularly, we first multiply divide the first Brillouin zone uniformly so that it contains a sufficiently large number of *k* points (5000 points in our calculations), and then do the sum of these points. The accuracy of this numerical procedure is ensured by requiring these subdivided regions small enough so that the integrand nearly becomes a constant within them. Moreover, the tight-binding wave function can be expressed as

$$|\Psi \left(k,j\right)\rangle =\sum_{j=1}^{N_{a}}(A_{j}|A_{j}\rangle +B_{j}|B_{j}\rangle ),$$

where $A_{j}$$\left(B_{j}\right)$ is the subenvelope functions on the A (B) sublattices which represents amplitude of the tight-binding functions $|A_{j}\rangle$ ($|B_{j}\rangle$), and $N_{a}$ is the number of atom in a unit cell. Since the wave functions of the initial and finial states are required to be normalized for computing the absorption coefficient, only the subenvelope functions will be needed. Here, we numerically solve the tight-binding Hamiltonian to obtain the energy and subenvelope functions (by applying a widely used Matlab code).

## 3. Results and Discussion

For a pristine AGNR, the band structure exhibits a band gap that is inversely proportional to the ribbon width [32]. In order to highlight the influence of the edge extension on the electronic properties of AGNRs, we introduce defects into a narrow ribbon (N = 7). It is worth mentioning that the current experiments for the edge-defect GNRs mainly focus on the narrow GNR [14,23]. Our numerical calculations for three different types of edge extension show that the edge bands occur in the vicinity of the Fermi level $E_{F}$ = 0. The valence and conduction edge bands retain the mirror symmetry as for the pristine systems. The main features of energy dispersion are sensitive to both *n* and *m*. These characteristics are in good agreement with the experimental measurements and previous theoretical predictions on the electronic properties of edge-extended GNRs [13,14,23,24,25]. The unique band structures are associated with the corresponding absorption spectra which we will discuss thoroughly next.

**Figure 2:**
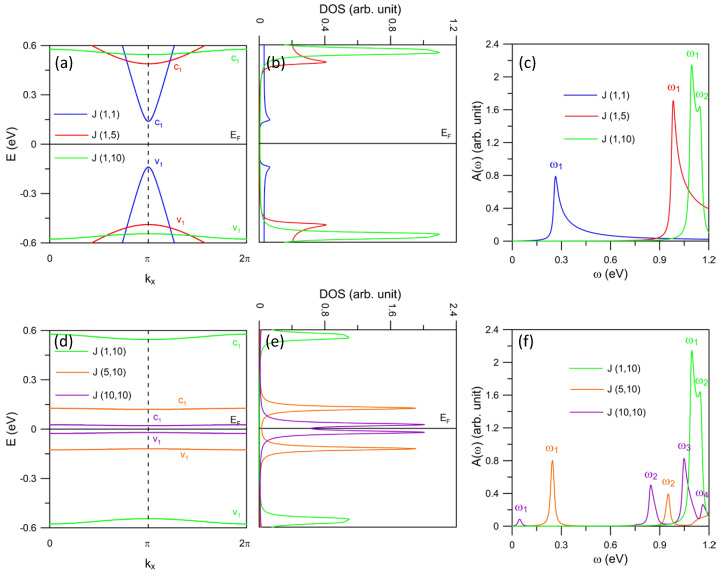
(color online) (**a**) Electronic band structure of J-(n,m) AGNRs with *n* = 1 and various chosen values of *m*, (**b**) the corresponding DOS and (**c**) the absorption spectra. Panels (**d**–**f**) illustrate similar plots for *m* = 10 and various *n*’s.

In order to investigate the roles played by *n* and *m* on the energy dispersion of the system, we first keep *n* fixed but change *m*, and then keep *m* fixed while altering the value of *n*. We find that the variation of *m* can greatly modify both the energy dispersion and the band gap while *n* is the dominant factor to tune the band gap. Note that, the change of band shape by varying *m* as well as the engineering of band gap by varying *n* are not significant in several cases. For the heterojunction J-(n,m), the band structures obtained by varying *n* and *m* are displayed in Figure 2a,d. Particularly, for *n* = 1, the increase of *m* gradually reshapes the ($v_{1}$, $c_{1}$) pair of the bands from parabolic to nearly-flat formations, as shown in Figure 2a. On the other hand, the size of the band gap possesses a proportional relationship with *n* for chosen *m*, referring to Figure 2d for *m* = 10. The substantial band features and their dependence on the lattice configuration are clearly reflected in the DOS. Figure 2b,e illustrate the DOS where the individual curves correspond to the energy bands with the same color. There exist the dominant peaks at the energies where the extrema of the valence and conduction bands or the nearly-flat bands are located. Consequently, these peaks are symmetric with respect to the Fermi level. Their amplitudes imitate the energy dispersion, i.e., the flat bands present predominant DOS features. Furthermore, the zero DOS in the vicinity of $E_{F}$ = 0 indicates the absence of electronic states within the band gap. The DOS spectrum is crucial for an extensive understanding of the optical excitations via the absorption intensity, according to the absorption function in Equation (2).

The ω-dependent absorption spectra exhibit unique peaks, as demonstrated in Figure 2c,f for various sets of (*n*, *m*). Each peak corresponds to a specific transition between the extreme values of the valence and conduction bands or the nearly-flat bands. The frequency of the optical threshold ($\omega_{1}$) reflects on the band gap. Meanwhile, its amplitude contains information regarding the state distribution and Hamiltonian matrix based on the velocity matrix elements in Equation (2). For example, the threshold frequency of J-(1,5) (red line) is higher than that of J-(1,1) (blue line), but lower than that of J-(1,10) (green line) (see Figure 2c). This is consistent with the band gap size relation for these three systems. This can also explain the difference in frequency of $\omega_{1}$ for J-(1,10), J-(5,10), and J(10,10) shown in Figure 2f. Moreover, the absorption peaks in the higher frequency range labeled as $\omega_{2}$, $\omega_{3}$, and $\omega_{4}$ for J-(5,10) and J(10,10) come from the transitions associated with the states at higher and deeper energies, such as $v_{2}$ and $c_{2}$ (not shown). These energy bands move towards the Fermi level for increasing *n*. Hence for AGNR, the absorption spectrum in the low-frequency region can be significantly enriched by the proper modification of the edge configuration.

The spectral intensity can be understood via the DOS. The transition between the occupied and unoccupied bands with higher-DOS is expected to yield stronger absorption intensity. This is generally true for most J-(n,m) systems, as illustrated in Figure 2c. Interestingly, this explanation fails for specific configurations of edge-extended AGNRs, such as the J-(5,10) and J-(10,10) (see the orange and purple lines in Figure 2e). These systems present relatively low threshold optical peaks though the corresponding DOS shown in Figure 2e is predominant. We observe that the optical transition is enhanced if the initial and final states belong to the parabolic bands with opposite slopes. However, it is suppressed for the nearly-flat bands. Our numerical calculations show that, for the J-(n,m) type, the Hamiltonian matrices of the systems possessing the nearly-flat edge bands contain special elements, which lead to the inconsequential velocity matrix elements. These elements are constructed from multiple interaction terms, which exhibit special real-space lattice symmetry resulting from the presence of edge defects. In particular, it is seen from Equation (1) that each Hamiltonian matrix element consists of the hopping interactions between a carbon atom and its nearest-neighboring ones. These hopping parameters are further accompanied by corresponding geometric phases, which describe both the inner and edge lattice structures of the system, such as the lattice constant and symmetry. Therefore, the Hamiltonian matrix elements of AGNRs will depend on various selected edge defects. In this way, both the inner-lattice periodicity and the edge-lattice periodicity can be fully built into the Hamiltonian in Equation (1). As a result, the associated velocity matrix elements in Equation (2) are infinitesimal. This can account for the extremely low threshold peaks of the systems with nearly-flat $v_{1}$ and $c_{1}$ edge bands. This unusual optical selection rule is attributed to the influence of edge-modification on the state distribution of the system.

**Figure 3:**
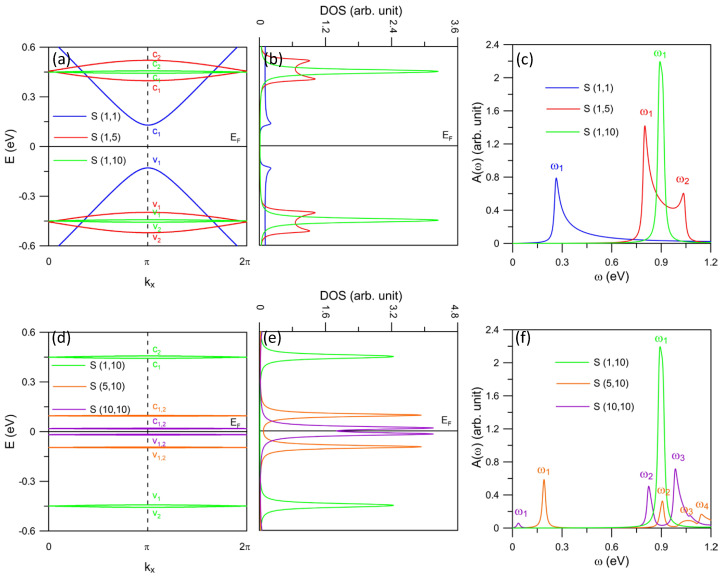
(color online) (**a**) Electronic structure of S-(n,m) AGNRs with *n* = 1 and various *m*’s. (**b**) The corresponding DOS and (**c**) absorption spectra. Panels (**d**–**f**) present similar plots for *m* = 10 and various *n*’s.

For the S-(n,m) AGNR, the change in energy dispersion with varying (n,m) is more clear compared with that of the J-(n,m). For a fixed *n*, the increase of *m* notably reduces the band gap within the conduction states (for example, $c_{1}$ and $c_{2}$) or valence states (for example, $v_{1}$ and $v_{2}$), as shown in Figure 3a. This forms the double-peaks of the DOS (red lines in Figure 3b) and also the absorption spectrum (red lines in Figure 3c) for the case S-(1,5). Actually, this behavior also exists in the S-(1,10) case, but with smaller spacing between the two components of the double-peaks (green lines). It is predicted that for the precise choice of (n,m), the double-peaks might merge together to form a single structure with enhanced amplitude. However, even for the cases of S-(5,10) and S-(10,10) when $v_{1}$ and $v_{2}$ (also $c_{1}$ and $c_{2}$) mostly overlap (see Figure 3d), the aforementioned optical selection rule results in suppression of the $\omega_{1}$ absorption intensity (see Figure 3f).

Another optical selection rule is realized for the S-(n,m) case, which disallows the $v_{1}\to c_{2}$ and $v_{2}\to c_{1}$ transitions. It is noticed that, the unoccupied and occupied bands belonging to the forbidden excitation channels exhibit the same slopes. Such a phenomenon is related to their wave functions, which determine the optical selection rule for the system. Explicitly, the dissimilarity between the number of zero modes of wave functions of the initial $|\Psi^{v}\left(k,i\right)\rangle$ and final $|\Psi^{c}\left(k,j\right)\rangle$ excitation states in Equation (2) in the cases S-(1,5) and S-(1,10) gives rise to certain forbidden optical transitions. In general, the S-(n,m) AGNR is subject to two different optical selection rules, i.e., (1) the suppression of velocity matrix elements associated with special H matrix elements due to the edge defect; (2) the correlation between the number of zero modes of the the initial and final wave functions for an excitation. The fact that the S-(n,m) AGNR is subject to two different optical selection rules is attributed to its special energy dispersion due to the asymmetry of two ribbon sides.

**Figure 4:**
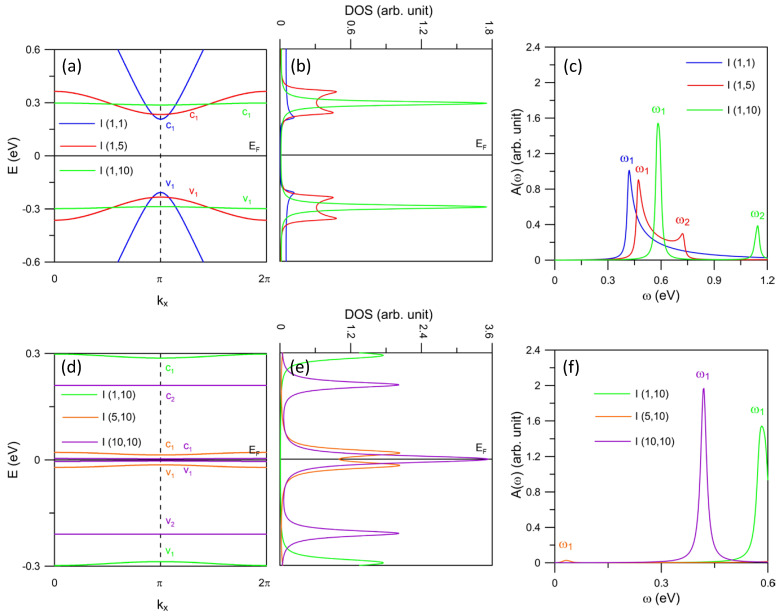
(color online) (**a**) Band structure of I-(n,m) AGNRs with *n* = 1 and various *m*’s. (**b**) The corresponding DOS and (**c**) absorption spectra for Γ = 10 meV. Panels (**d**–**f**) present similar plots for *m* = 10 and various *n* values.

The edge-extended AGNRs with I-(n,m) configuration present a distinctive energy dispersion and absorption spectra. In Figure 4a, the reformation of band structure follows the same way as for the J-(n,m) and S-(n,m) systems. Though, the energy spacing between the first ($v_{1}$, $c_{1}$) and second ($v_{2}$, $c_{2}$) pairs is quite large throughout the first Brillouin zone, which we refer to the purple lines in Figure 4d for I-(10,10). Furthermore, there exist the double-peak structures for I-(1,5), coming from the multi-extreme points of $v_{1}$ and $c_{1}$ at $k_{x}$ = 0, π, 2π. This is different from the double-peak initiation of S-(1,5) where the two component peaks correspond to two separated energy bands.

Interestingly, the breakdown of the conventional optical selection rules discussed above is found in the I-(n,m) AGNRs. It is straightforward that the ($v_{1}$, $c_{1}$) pair becomes more flat for I-(1,5) (red lines in Figure 4a) than for I-(1,1) (blue lines). Moreover, the corresponding DOS is higher for I-(1,5) (see Figure 4b). However, as illustrated in Figure 4c, the threshold absorption peaks of I-(1,5) are lower than that of I-(1,1). We observe that the extraordinary H matrix elements originated from the unique lattice configuration of I-(n,m) leading to such unpredicted optical features. Explicitly, such elements are associated with the defect-enabled special symmetry in the lattice structure so that their corresponding velocity matrix elements in Equation (2) become unique for defect-specific bands. Additionally, the optical excitations between the nearly-flat bands give rise to substantial absorption peaks, for example, the $v_{2}\to c_{2}$ of the I-(10,10) system (purple line in Figure 4f). These behaviors are contrary to the cases of J-(n,m) and S-(n,m) discussed above, as they do not obey the aforementioned selection rule, which is based on the relationship between the wave functions. We note that both the optical selection rules are only applicable for the first pairs of edge bands. Our observation of such a singular optical characteristic is considered as a hint for the possible explanation of similar results from spectroscopy measurements.

Now we turn our attention to investigating the role played by the type of edge-extension in the electronic and absorption spectra of AGNRs. This can be understood via the comparison between the three different types of edge defects in terms of band structure, DOS, and absorption spectra. Due to special lattice asymmetric arrangement, the S-(n,m) type of edge defect can adjust the band degeneracy, while this is absent for the J-(n,m) and I-(n,m) configurations. This explains the relatively higher DOS for S-(n,m). The low-frequency absorption spectra present comparable absorption intensities regardless of the difference in DOS. In general, proper modification of (*n*, *m*) can notably alter both the optical threshold and absorption intensity of the edge-extended AGNRs. The theoretical prediction offered in this work provides useful information in the search for suitable materials for possible technology applications.

## 4. Concluding Remarks

In summary, we have investigated the rich electronic and optical properties of edge-extended AGNRs. The tight-binding Hamiltonian was constructed for each ribbon system with specific boundary conditions to study the band structures and DOS. The absorption spectra were computed from the absorption function. We found that these essential properties of AGNRs are remarkably enriched by modification of the edge states. The flat bands, band gap variation, and unique features of the DOS and absorption spectra were presented for various types and periods of the edge extension. Moreover, the unusual optical selection rule was demonstrated via the unusual transition channels associated with the flat bands for selected edge-modified systems. We showed that proper modification of (*n*, *m*) can significantly alter both the optical threshold and absorption intensity of the edge-extended AGNRs. Our theoretical prediction opens an opportunity for extensive comprehension of the influence of edge defects on the electronic and optical properties of AGNRs.

Possible applications of nanoribbons could be in sensing and nano-imaging in very large wavelength range, extending from visible to infrared frequencies [38,39]. Additionally, plasmons have been studied considerably in graphene [40,41] and other bulk Dirac materials. Now, after almost two decades of unrelenting perseverance [42,43], the plasmon dynamical properties of low-dimensional structures are now well understood with the help of many-particle theory. The polarization function can now be accurately calculated and plays a key role in calculations of the dielectric function, which can in turn be used for determining plasmon dispersion and screening. Our results, on the other hand, reveal unique optical properties capable of generating significant interest. These nanomaterials are capable of hosting extremely strong light–matter interactions as a consequence of the enhanced excitonic effect in two dimensions. Therefore, it is crucial to fully understand the excitons to unlocking the potential of these nanoribbons for future photonic and optoelectronic devices. Finally, possible devices include optical modulators, excitonic light emitting diodes, lasers, and coupling in an optical cavity.
